# Malaria Related Neurocognitive Deficits and Behavioral Alterations

**DOI:** 10.3389/fcimb.2022.829413

**Published:** 2022-02-22

**Authors:** Pamela Rosa-Gonçalves, Flávia Lima Ribeiro-Gomes, Cláudio Tadeu Daniel-Ribeiro

**Affiliations:** ^1^ Laboratório de Pesquisa em Malária, Instituto Oswaldo Cruz, Fundação Oswaldo Cruz (Fiocruz), Rio de Janeiro, Brazil; ^2^ Centro de Pesquisa, Diagnóstico e Treinamento em Malária, Fiocruz and Secretaria de Vigilância em Saúde, Ministério da Saúde, Rio de Janeiro, Brazil; ^3^ Laboratório de Biologia, campus Duque de Caxias, Colégio Pedro II, Duque de Caxias, Brazil

**Keywords:** severe malaria, non-severe malaria, neurocognitive deficits, behavioral alterations, murine malaria

## Abstract

Typical of tropical and subtropical regions, malaria is caused by protozoa of the genus *Plasmodium* and is, still today, despite all efforts and advances in controlling the disease, a major issue of public health. Its clinical course can present either as the classic episodes of fever, sweating, chills and headache or as nonspecific symptoms of acute febrile syndromes and may evolve to severe forms. Survivors of cerebral malaria, the most severe and lethal complication of the disease, might develop neurological, cognitive and behavioral sequelae. This overview discusses the neurocognitive deficits and behavioral alterations resulting from human naturally acquired infections and murine experimental models of malaria. We highlighted recent reports of cognitive and behavioral sequelae of non-severe malaria, the most prevalent clinical form of the disease worldwide. These sequelae have gained more attention in recent years and therapies for them are required and demand advances in the understanding of neuropathogenesis. Recent studies using experimental murine models point to immunomodulation as a potential approach to prevent or revert neurocognitive sequelae of malaria.

## Introduction

Malaria, an important parasitic infectious disease since antiquity, remains a significant public health problem, being responsible for estimated 229 million cases and 409.000 deaths worldwide annually (World Health Organization, 2020). It is caused by protozoa of the genus *Plasmodium* and is transmitted by the bite of the female *Anopheles* mosquito. There are eight species causing human malaria: *P. falciparum, P. vivax, P. malariae, P. ovale curtisi* and *P. ovale wallikeri* (Sutherland et al., 2010), *P. knowlesi, P. cynomolgi* and *P. simium*, the last three being simian parasites responsible for zoonotic infections (Chin et al., 1965; Singh et al., 2004; Ta et al., 2014; Brasil et al., 2017a), and *P. falciparum*, which accounts for the great majority of cases and severe forms of the disease (Cox, 2010). In the early stages of infection, malaria may present with nonspecific symptoms of febrile syndromes (including nausea and diarrhea) before the emergence of the classic triad (fever, chills, sweating), often associated with headache. The disease may evolve, in case of falciparum malaria, to its severe forms such as cerebral malaria (CM), severe acute respiratory syndrome, severe malarial anemia, among others (Ashley et al., 2018). Although severe anemia is the most common complication of the disease, CM is the deadliest one, affecting mainly children up to 5 years old, pregnant and non-immune individuals (tourists and military) from non-endemic areas (Porta et al., 1993; Ashley et al., 2018; Ghazanfari et al., 2018).

The neuropathogenesis of CM involves brain intravascular sequestration of *P. falciparum*-parasitized red blood cells occurring through the expression of parasite-encoded *P. falciparum* erythrocyte membrane protein-1 (PfEMP-1) in protuberances of the infected red-cell surface that interacts with host adhesion receptors on endothelium, contributing to the inflammatory process (Lau et al., 2015). These events participate in the generation of blood flow impairment, endothelial dysfunction, intravascular coagulation, vascular occlusion, cerebral hypoxia, microglial activation, astrogliosis, disruption of the blood-brain barrier and neurotoxicity (Medana et al., 2002; Taylor et al., 2004; Dorovini-Zis et al., 2011; Schiess et al., 2020).

Post treatment long-term neurocognitive deficits, including in multiple areas of cognitive function, and behavioral alterations^1^ are related to severe malaria, mainly CM (Bangirana et al., 2014; Ssenkusu et al., 2016; Conroy et al., 2019a). Nevertheless, cognitive deficits and behavioral alterations have also been associated to the non-severe presentations of the disease (Vitor-Silva et al., 2009; Fink et al., 2013; Tapajós et al., 2019).

The aim of this mini review is to provide an overview on the state of art and on the available knowledge in the literature on neurocognitive and behavioral damage associated to malaria. There are few articles on the subject and some of them have limitations, due to involving human beings, having been carried out in areas lacking adequate infrastructure, or having critical design or methodological aspects. Although a detailed analysis on the structure of the cited articles is out of this mini review scope, we have tried to indicate points of concern deserving attention whenever appropriate.

## Neurocognitive and Behavioral Sequelae of Severe Malaria

**Figure 1:**
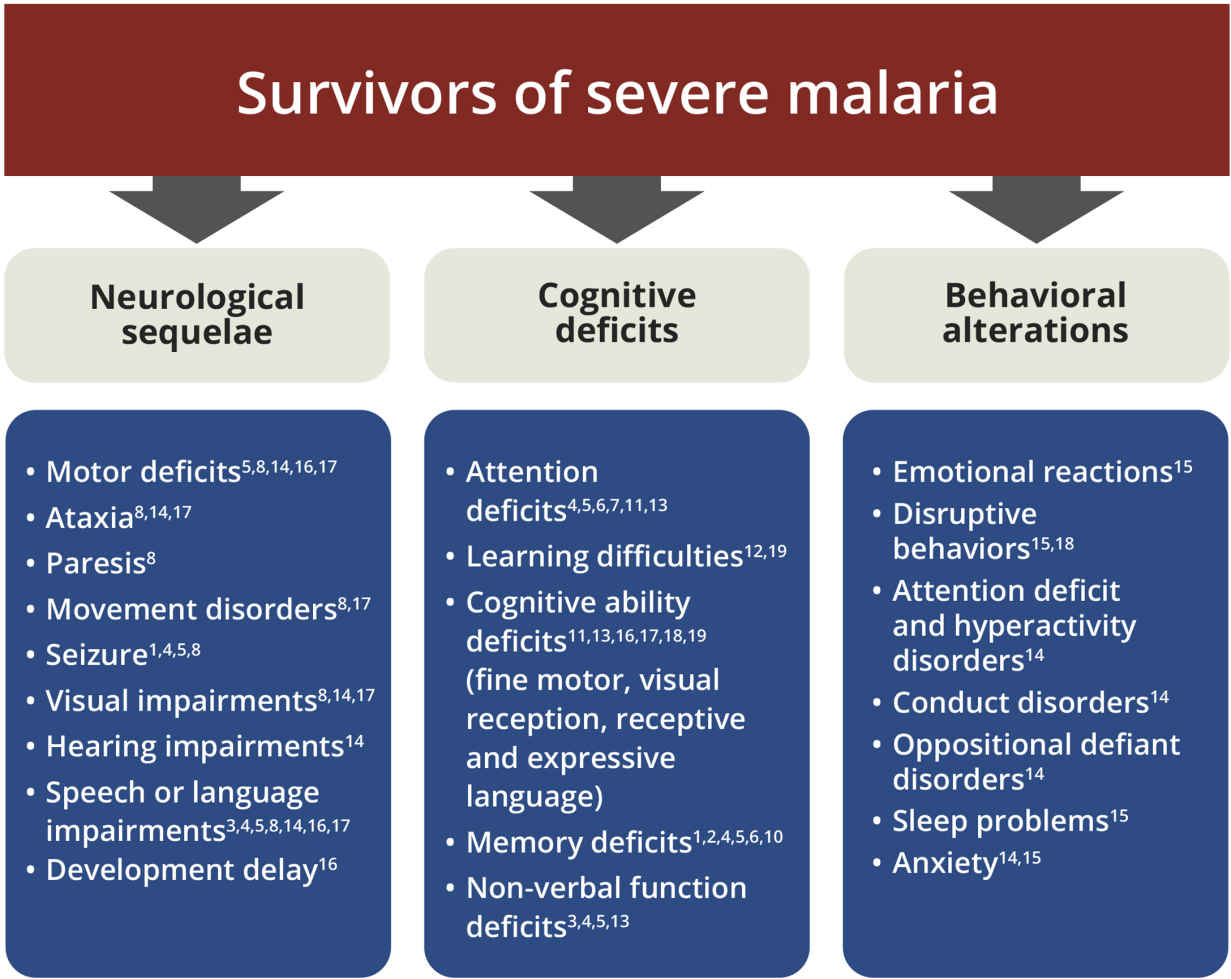
Spectrum of neurologic, cognitive and behavioral sequelae among survivors of severe malaria. List of main impairments after severe malaria. ^1^ Varney et al. (1997); ^2^ Richardson et al. (1997); ^3^ Carter et al. (2005a); ^4^ Carter et al. (2005b); ^5^ Idro et al. (2006); ^6^ Boivin et al. (2007); ^7^ John et al. (2008); ^8^ Birbeck et al. (2010); ^9^ Bangirana et al. (2011); ^10^ Laverse et al. (2013); ^11^ Bangirana et al. (2014); ^12^ Peixoto and Kalei (2013); ^13^ Bangirana et al. (2016); ^14^ Idro et al. (2016); ^15^ Ssenkusu et al. (2016); ^16^ Brim et al. (2017); ^17^ Conroy et al. (2019b); ^18^ Langfitt et al. (2019); ^19^ Ouma et al. (2021).

Individuals with CM treated with artesunate, a potent antimalarial treatment that promotes a rapid decline in parasitemia and recovery (Dondorp et al., 2010), still progress to death in about 15-25% of cases (Bruneel, 2019). Unfortunately, approximately 25% of survivors develop neurocognitive and behavioral sequelae (Bruneel, 2019). Several studies have associated the most varied neurological sequelae, cognitive deficits and behavioral alterations, as well as predisposition to mental disorders in children (Carter et al., 2005a; Carter et al., 2005b; Carter et al., 2006; Idro et al., 2006; Boivin et al., 2007; Birbeck et al., 2010; Bangirana et al., 2011; Idro et al., 2016; Ssenkusu et al., 2016; Brim et al., 2017; Langfitt et al., 2019) and adults (Richardson et al., 1997; Varney et al., 1997; Laverse et al., 2013; Peixoto and Kalei, 2013) with CM (**Figure 1**).

Among children up to five years old, a greater risk for the development of impairment in motor skills, receptive and expressive language, visual reception and social interaction has been identified (Brim et al., 2017; Ouma et al., 2021). Multiple seizures, prolonged coma and intracranial hypertension during CM appear to be related to the risk of neurological sequelae including epilepsy (Idro et al., 2004; Idro et al., 2006; Kariuki et al., 2011). CM does also result in deficits in multiple cognitive domains such as attention and associative memory (Bangirana et al., 2014) and increased risk of mental health disorders (Idro et al., 2016).

The dynamics of malaria deficits can be affected by several factors, such as infection at different stages of neurodevelopment, number of previous malaria episodes, methodology used to assess the neurocognitive outcomes, and follow-up time. Although some neurological sequelae of CM in children, such as hypotonia, cortical blindness, tremors and aphasia recover over time (Boivin et al., 2007; John et al., 2008; Dondorp et al., 2010), other such as hemiplegia, paresis, seizures, language deficits and, especially, cognitive impairment in memory, non-verbal function and behavioral alterations may persist (Carter et al., 2006; Birbeck et al., 2010; Oluwayemi et al., 2013; Severe Malaria, 2014) (**Table 1**) for up to nine years after an episode of CM (Carter et al., 2005a).

**Table 1:** Detection and persistence of neurocognitive and behavioral alterations in children with cerebral malaria.

Outcomes	Impairments at discharge	Months*			n	Reference
		6	12	24		
**Neurologic sequelae**						
Neurologic deficits	**++++++**	**+**	**+**	**+**	232	Conroy et al. (2021)
Motor	**+++++**		**+**		173	Idro et al. (2016)
	**+++++**			**+**	225	Conroy et al. (2019b)
Movement disorders	**+**			**+**	225	Conroy et al. (2019b)
Visual	**++**		**-**		173	Idro et al. (2016)
	**++**			**-**	225	Conroy et al. (2019b)
Hearing	**+**		**-**		173	Idro et al. (2016)
Speech and/or language	**++**		**+**		131	Oluwayemi et al. (2013)
	**++**		**-**		173	Idro et al. (2016)
	**+++**			**+**	225	Conroy et al. (2019b)
Paresis	**+**		**+**		131	Oluwayemi et al. (2013)
Seizure	**+**		**+**		131	Oluwayemi et al. (2013)
Hyporeflexia or Babinski sign	**+++**			**-**	225	Conroy et al. (2019b)
Ataxia and/or gait problems	**+++**		**-**		173	Idro et al. (2016)
	**++++**			**-**	225	Conroy et al. (2019b)
**Cognitive deficits**						
MSEL	**+**	**+**	**+**		80	Bangirana et al. (2016)
KABC or MDAT	**+**	**+**	**+**		85	Langfitt et al. (2019)
RBMTC				**+**	152	Carter et al. (2006)
Memory deficits	**+**		**+**	**+**	131	Oluwayemi et al. (2013)
Attention	**+**	+**		**++++**	38	John et al. (2008)
**Behavioral alterations**						
Increased risk of expression of mental disorders	**-**	**-**	**+++**	**-**	173	Idro et al. (2016)
Behavioral dysfunctions	**+**	**+**	**+**	**+**	100	Ssenkusu et al. (2016)

*Approximate time of follow up; **not statistically significant; n, number of individuals; +, 0,1-4,99% or detected; ++, 5-9,99%; +++, 10-14,99%; ++++, 15-19,99%; +++++, 20-24,99%; ++++++, >25%; -, not detected; blank spaces, not accessed; MSEL, Mullen Scales of Early Learning; KABC, Kauffman Assessment Battery for Children; MDAT, Malawi Developmental Assessment Tool; RBMTC, Rivermead Behavioural Memory Test for Children; Some studies establish the percentage of damage among individuals who developed any neurocognitive impairment after malaria, or among CM survivors; other displayed the results in z scores that are not interpreted quantitatively as percentage values. Whenever there was a difference in the z score, it was interpreted qualitatively, as a detected or undetected outcome.

Long-term cognitive ability deficits are also observed in children after severe malarial anemia (Bangirana et al., 2014; Conroy et al., 2019a). Severe anemia might affect the overall cognitive ability related to neurocognitive domains for fine motor scales, visual reception, receptive and expressive languages (Bangirana et al., 2014; Bangirana et al., 2016). Furthermore, acute kidney injury, a complication of severe malaria, is a risk factor for long-term neurocognitive impairment and behavioral problems in children with severe malarial anemia and cerebral malaria in overall cognitive ability (Conroy et al., 2019b), socio-emotional (aggressive behavior) and executive functions (Hickson et al., 2019).

Murine models have made invaluable contributions to malaria research (Lou et al., 2001). Different strains of mice infected by different species of plasmodia reproduce different forms of the disease depending on the combination of parasite and host, exhibiting susceptibility or resistance to severe forms. Neurologic, cognitive and behavioral deficits observed in malaria patients are reproduced in murine model of experimental CM (ECM). Infection of C57BL/6 and Swiss mice with *Plasmodium berghei*, ANKA strain (Grau et al., 1990; Martins et al., 2009; Martins et al., 2016), or Swiss mice with the lethal strain of *P. yoelli* (Li et al., 2001; Reis et al., 2010) are also models of ECM.

C57BL/6 mouse model infected with *P. berghei* ANKA is the classic reference for the study of ECM (Grau et al., 1990). The absence of a protein analogous to PfEMP-1, which binds to ligands overexpressed by influence of inflammatory cytokines on vessel endothelium in *P. berghei*, contributes to a limited sequestration of parasitized erythrocytes in the murine cerebral microvasculature. This model has, nonetheless, many characteristics that resemble human CM (Brian De Souza and Riley, 2002; De Niz and Heussler, 2018; Ghazanfari et al., 2018). Three days after infection (D3), low levels of parasitemia are measured, which gradually increase during the course of the infection. At D4, a slight adhesion of few leukocytes to the cerebral microvasculature may begin, with minimal presence of edema restricted to some punctual areas of the brain (Potter et al., 2006). Clinical neurological signs are identified from D5 on, with the establishment of a clear neurological syndrome at D6, including vascular inflammation, endothelial activation, rupture of the blood-brain barrier, edema and punctiform hemorrhage in parts of the brain, in addition to the set of neurocognitive sequelae and behavioral alterations assessable in behavioral tests in the acute phase and after recovery from ECM (Brian De Souza and Riley, 2002; Hunt et al., 2006; Potter et al., 2006; Desruisseaux et al., 2008; Dai et al., 2010; Reis et al., 2010; Lacerda-Queiroz et al., 2010; Martins et al., 2010; De Miranda et al., 2011; Reverchon et al., 2017; Ghazanfari et al., 2018; Lima et al., 2020). Generally, between days 6 and 9 the animals die (Martins et al., 2010).

During acute ECM, C57BL/6 mice infected with *P. berghei* ANKA exhibit motor impairment. In the course of acute infection, mice do also present cognitive deficits in object recognition test of working memory (Desruisseaux et al., 2008), anxiety-like behavior in elevated plus maze test (De Miranda et al., 2011) and depressive-like behavior in tail suspension and forced swim tests (Lima et al., 2020). Nonetheless and most important, specific neurological sequelae, including poor performance in balance beam test to evaluate motor coordination, are clearly present as sequelae after treatment of ECM (Dai et al., 2010; Reis et al., 2010). Cognitive deficits in multiple memory impairment (habituation, aversive and recognition memories) and behavioral alterations (depressive-like behavior) are also reported after CM (Reis et al., 2010; Lima et al., 2020).

Some experimental murine models, non-susceptible to the development of experimental CM, are useful to reproduce severe forms of non-cerebral malaria, for instance: *P. berghei* NK65 infected C57BL/6 mice are used as an experimental model to investigate acute respiratory distress syndrome in malaria (Van Den Steen et al., 2010; Scaccabarozzi et al., 2018), while BALB/c mice infected with *P. berghei* ANKA compose a model of severe anemia and severe placental malaria (Neres et al., 2008; De Niz and Heussler, 2018). However, cognitive deficits were not registered in most of these experimental murine models of severe forms other than cerebral malaria (Reis et al., 2010; Freeman et al., 2016). Only a minor occurrence of neurological alterations of locomotor activity and autonomic function was observed in BALB/c mice infected with *P. berghei* ANKA (Reis et al., 2010), as well as cognitive injury in the offspring of infected pregnant BALB/c mice (McDonald et al., 2015).

## Cognitive and Behavioral Sequelae of Non-Severe Malaria

Cognitive and behavioral deficits are also observed after episodes of non-severe malaria in individuals from diverse *P. falciparum* and *P. vivax* endemic areas (Fernando et al., 2003; Vitor-Silva et al., 2009; Thuilliez et al., 2010; Fink et al., 2013; Vorasan et al., 2015; Brasil et al., 2017b; Tapajós et al., 2019). The reported effects have been found in individuals who had uncomplicated febrile episodes of malaria (Vitor-Silva et al., 2009; Vorasan et al., 2015; Brasil et al., 2017b; Tapajós et al., 2019) and even asymptomatic infections (Al Serouri et al., 2000; Nankabirwa et al., 2013; Clarke et al., 2017).

Impairments in the school performance associated with language and logical reasoning in mathematics have been identified in children from Brazil and Sri Lanka after non-severe malaria (Fernando et al., 2003; Vitor-Silva et al., 2009). In addition, children with more than five episodes performed less well than children who had up to three febrile attacks (Fernando et al., 2003). Additional studies with different ranges in the number of past episodes of malaria can confirm and reinforce the observation of a quantitative effect of malaria on the cognitive-behavioral performance of infected children. School performance was evaluated during a nine-month follow-up (Vitor-Silva et al., 2009). Tapajós et al. (2019) clearly demonstrated that an episode of non-severe malaria, caused by *P. vivax*, was a determining factor – independent of the socioeconomic status, family stimulus and child’s health status – in triggering cognitive impairment in children aged two to seven years. In Zambia and Uganda, episodes of malaria (with or without anemia) were associated with impaired cognitive ability in children (Fink et al., 2013; Boivin et al., 2016). Fink et al. (2013) point out that children’s exposure to malaria was associated with reduced socio-emotional development. It is worth mentioning that the effect size was not shown in this study, which did not evaluate individual infection rates, but rather used national data.

On the other hand, classic murine models of non-severe malaria as C57BL/6 mice infected with *P. chabaudi* and Swiss with *P. yoelii* (non-lethal) were unable to reveal the presence of cognitive impairment (Reis et al., 2010; Guha et al., 2014). Behavioral alterations, such as anxiety-like behavior and social interaction deficits, are observed during the course of non-severe malaria (D9) in C57BL/6 mice infected by *P. chabaudi adami* (Guha et al., 2014). These alterations may be, however, at least partially due to the inflammatory state of animal during the course of the ongoing infection. In this model, infection also predisposes to enhanced post-stress anxiety-like responses when non-severe malaria occurs at a young age (Guha et al., 2020).

More recently, De Sousa et al., (2018) infected C57BL/6 mice with *P. berghei* ANKA (the classic model of experimental CM), treating mice at D4, before any neurological manifestation of CM. This model proved to be useful for detecting cognitive sequelae and late (D92) behavioral alterations following (82 days after parasite clearance) a single episode of non-severe malaria (De Sousa et al., 2021).

## Therapeutic Approaches to Neurocognitive and Behavioral Sequelae in Malaria

To date, no effective therapy for the cognitive and behavioral sequelae of malaria is available. The lack of an effective therapy is associated with an incomplete understanding of the mechanisms involved in pathogenesis of cognitive and behavioral sequelae, especially in non-severe malaria. This knowledge is important to the development of rational therapies, directed to specific targets.

While several clinical studies to treat CM appeared promising for focusing on specific targets of pathogenesis, as sequestration of parasitized erythrocytes in the microvasculature, inflammation, coagulation, endothelial function and oxidative stress, they have not been successful in reducing neurologic sequelae (Lell et al., 2010; Mwanga-Amumpaire et al., 2015; Schiess et al., 2020). Some approaches have even aggravated these sequelae (Warrell et al., 1982; Van Hensbroek et al., 1996; Akech et al., 2006).

In addition, many studies investigating adjuvant therapies in CM do not assess the effects on neurocognitive sequelae. Although antimalarials may not be considered an approach to treat these sequelae, Conroy et al. (2021) recently showed that treatment of severe malaria with derivatives of artemisinin in children resulted in a significant reduction in mortality and in neurological deficits, associated with improvement in long-term behavioral alterations compared to quinine treatment. The absence of effect on overall cognition, attention and memory but a specific improvement in the executive function impairment, revealed by the BRIEF (Behaviour Rating Inventory of Executive Function) test observed by the authors, suggest that antimalarials may have distinct impacts on different categories of sequelae. Treatment of cognitive deficits remains a challenge. Some studies showed an expected positive effect of preventive malaria regimens on cognitive function of chronically exposed individuals in endemic areas (Cohee et al., 2020).

Several *in vivo* experimental studies have performed interventions – before infection or associated with antimalarial treatment – in an attempt to improve clinical response, increase survival and reduce neurocognitive and behavioral impairments (Varo et al., 2018; Gul et al., 2021). Serghides et al. (2014) got compelling effects of rosiglitazone, a PPARɣ agonist, as an adjuvant treatment of ECM. This treatment ameliorated recognition memory, reduced endothelial activation and oxidative stress, and increased neurotrophic factors (Varo et al., 2018). Currently the neurocognitive impact of this approach on CM is being accessed in a clinical trial. Lima et al. (2020) obtained promising results administrating mesenchymal stem cells (MSC) as adjuvant therapy to chloroquine at day 6 of infection, for ECM. Treatment with a single dose of MSC has protected infected mice against vascular damage and improved depressive-like behavior. Witschkowski et al. (2020) reported that bacillus Calmette-Guérin (BCG) vaccination given 10 or 30 days before infection protected mice from developing clinical symptoms of ECM and improved survival associated with immunosuppressive mechanisms. De Miranda et al., (2017) registered that treatment with MK801, a N-methil-D-aspartate (NMDA) receptor antagonist, before signs of ECM (from D3 to the end of antimalarial chloroquine treatment) prevented long-term memory impairment and depressive-like behavior.

Since diagnosis of CM does occur when the patient’s condition is severe, the period required for the introduction of the treatment may not be sufficient for its effectiveness and the adjuvant approaches quoted above may have a very restricted therapeutic window (Idro et al., 2010). In view of these limitations, emphasis should be given to studies approaching the treatment of already established neurocognitive impairments. Neuroinflammation is a hallmark of neurocognitive and behavioral impairments of malaria and may result from peripheral inflammation (Sankowski et al., 2015). Therefore, immunomodulatory approaches hold promise for alleviating or stopping long-term damage.

Cognitive rehabilitation of severe malaria survivors through computerized training has shown an immediate improvement in some neuropsychological and behavioral parameters in children in Africa (Bangirana et al., 2009; Boivin et al., 2019). However, this approach in patients who have survived severe malaria may be limited by the need of specific diagnosis, long-term follow-up and information on how long this effect will last. Another limitation to the use of such approach is that it requires specific computer systems, often unavailable in endemic areas of developing countries (Boivin et al., 2019).

In the last few years, evidence has accumulated pointing to the ability of immune stimuli to modulate neurogenesis, synaptic plasticity, in addition to cognitive and behavioral function (Xia et al., 2014; Li et al., 2015; Li et al., 2016; Yang et al., 2016; Qi et al., 2017; Yang et al., 2020; De Sousa et al., 2021). These findings support the concept of consistent communication and interrelationship between the nervous and immune systems, as plastic and cognitive systems (Jerne and Cocteau, 1984; Pert et al., 1985; Cohen, 1992; Sawada et al., 1993; Josefsson et al., 1996; Amenta et al., 2002; McKenna et al., 2002; Daniel-Ribeiro and Martins, 2017) and the use of immunomodulation as a promising approach in the treatment of neurocognitive and behavioral sequelae of infectious diseases.

In this context, our research group has been investigating the effect of immune stimuli on neurocognition. Active immunization of both healthy and *P. berghei* ANKA infected C57BL/6 mice using different integrated stimuli (elicitors of the Th2 response) improved recognition memory in homeostasis, recovered late deficits in cognitive function and inhibited the manifestation of an anxiety-like phenotype caused by a unique episode of non-severe malaria (De Sousa et al., 2021).

## Conclusions

In summary, accumulating evidence has revealed that malaria might lead to significant global cognitive impairment and behavioral alterations, even after non-severe episodes, that might persist for years. Neurological sequelae, however, seem to be restricted to severe malaria. These neurocognitive and behavioral sequelae of malaria lack specific and effective treatment. Results obtained in a few well conducted recent studies highlight the potential of immunomodulation as a cognitive enhancement strategy to treat cognitive and behavioral sequelae of malaria.

## Author Contributions

PR-G drafted the manuscript and PR-G, FR-G and CD-R reviewed, edited and prepared its final version. All authors read and approved the submitted version.

## Funding

FR-G and CD-R are supported by CNPq, Brazil, through a Productivity Research Fellowship, and CD-R is a “*Cientista do Nosso Estado”* by *Faperj*. The *Laboratório de Pesquisa em Malária, Instituto Oswaldo Cruz (IOC), Fiocruz* is an Associated Laboratory of the *Instituto Nacional de Ciência e Tecnologia (INCT)* in Neuroimmunomodulation supported by the CNPq, and of the Rio de Janeiro Neuroinflammation Network financed by Faperj.

## Conflict of Interest

The authors declare that the research was conducted in the absence of any commercial or financial relationships that could be construed as a potential conflict of interest.

## Publisher’s Note

All claims expressed in this article are solely those of the authors and do not necessarily represent those of their affiliated organizations, or those of the publisher, the editors and the reviewers. Any product that may be evaluated in this article, or claim that may be made by its manufacturer, is not guaranteed or endorsed by the publisher.

## Footnotes

1 Neurologic sequelae: consist of functional loss in motor, speech, vision, and hearing domains, as well as epilepsy; Neurocognitive deficits: a neurological and/or cognitive impairment involving specific brain-located neural phenomena; Cognitive deficits: decrease in skill related to broad sense of consciousness (how one perceives, remembers things, judges oneself); Behavioral alterations: behavior changes, including inattentiveness, hyperactivity, impulsive/ aggressive behaviors and risk of manifestation of mental health disorders.
